# Multiparameter RNA and Codon Optimization: A Standardized Tool to Assess and Enhance Autologous Mammalian Gene Expression

**DOI:** 10.1371/journal.pone.0017596

**Published:** 2011-03-03

**Authors:** Stephan Fath, Asli Petra Bauer, Michael Liss, Anne Spriestersbach, Barbara Maertens, Peter Hahn, Christine Ludwig, Frank Schäfer, Marcus Graf, Ralf Wagner

**Affiliations:** 1 Geneart AG, BioPark, Regensburg, Germany; 2 Molecular Microbiology and Gene Therapy Unit, Institute of Medical Microbiology and Hygiene, University of Regensburg, Regensburg, Germany; 3 QIAGEN GmbH, Hilden, Germany; University of Edinburgh, United Kingdom

## Abstract

Autologous expression of recombinant human proteins in human cells for biomedical research and product development is often hampered by low expression yields limiting subsequent structural and functional analyses. Following RNA and codon optimization, 50 candidate genes representing five classes of human proteins – transcription factors, ribosomal and polymerase subunits, protein kinases, membrane proteins and immunomodulators – all showed reliable, and 86% even elevated expression. Analysis of three representative examples showed no detrimental effect on protein solubility while unaltered functionality was demonstrated for JNK1, JNK3 and CDC2 using optimized constructs. Molecular analysis of a sequence-optimized transgene revealed positive effects at transcriptional, translational, and mRNA stability levels. Since improved expression was consistent in HEK293T, CHO and insect cells, it was not restricted to distinct mammalian cell systems. Additionally, optimized genes represent powerful tools in functional genomics, as demonstrated by the successful rescue of an siRNA-mediated knockdown using a sequence-optimized counterpart. This is the first large-scale study addressing the influence of multiparameter optimization on autologous human protein expression.

## Introduction

Heterologous expression of recombinant proteins is an indispensable process in modern biotechnology and biomedicine. *E. coli* is the preferred host for protein production due to its fast growth, easy handling, inexpensive culturing and well-studied genetics. However, besides the lack of posttranslational modifications or a suitable environment for membrane proteins, *E. coli*-mediated expression is often associated with protein misfolding or aggregation [1], imposing restrictions on large-size or oligomeric proteins.

To overcome these limitations, the repertoire of expression systems for recombinant proteins was extended to gram-positive bacteria, yeast, filamentous fungi, insect cells and plants [2]–[4]. Nevertheless, non-mammalian cells' inability to synthesize authentic human glycoproteins finally directed endeavors towards improving mammalian expression systems to fulfill the structural and functional quality requirements for downstream applications. Accordingly, 70% of recombinant protein pharmaceuticals and most proteins used for vaccination, human therapy or diagnostics are currently produced in mammalian cells [5]. In particular, cell lines such as CHO or HEK293 have become golden standards for high-yield production of functional recombinant human proteins.

However, even in autologous hosts, transcriptional silencing, mRNA destabilization, alternative splicing, premature polyadenylation, or inefficient translation often compromise protein expression. Although sometimes solved by engineering the expression host (e.g. providing rare tRNA pools [6]) or using improved expression cassettes with strong or tissue-specific promoters, most of these problems are gene-specific, requiring direct modification of the coding sequence.

Several DNA- or mRNA-based sequence motifs apparently play a decisive role in modulating gene expression. Whereas UpA-dinucleotides, preferred targets of endoribonuclease cleavage, seem to be critical for mRNA stability [7], CpG-dinucleotides provide hot-spots for mutations [8] and were implicated in methylation-dependent gene silencing [9]. In contrast, the intragenic CpG-content of transgenes was reported to directly correlate with *de novo* transcription [10]. AU-rich (ARE)-elements in the 3′ untranslated region of mRNAs are well-studied determinants of mRNA instability [11], [12], and some more complex AU-rich, repressive sequence-motifs identified in certain viral RNAs must be eliminated to allow independent mammalian expression of such genes [13]–[16].

Instead of identifying and eliminating such motifs, the same effect can be achieved by adapting the codon usage of these AT-rich viral genes to the more GC-rich codon preferences of mammalian genes. Due to the degeneracy of the genetic code, the use of synonymous codons for defined amino acids differs in each organism. Indeed, the strategy of using synonymous codons while maintaining the original protein sequence proved particularly successful in HIV research, increasing the stability of certain mRNAs by orders of magnitude [16], [17].

Several studies have proven the immense impact of codon choice on gene expression in mammalian cells [18], [19]. In particular, non-mammalian gene expression in mammalian hosts was significantly enhanced by substituting rare codons with more frequent ones [20]–[22]. Besides inter-species variations, codon usage even differs among human tissue cells [23] and mammalian housekeeping genes are usually associated with higher GC-content than low-expressing genes [24]. Recently, differences in tissue-specific expression of individual tRNA species and the relative abundance of tRNA-isoacceptors [25] were described to strongly correlate with the codon usage of genes highly expressed in specific tissues.

Such findings strongly suggest that a comprehensive optimization strategy involving simultaneous modulation of multiple sequence parameters might be the best solution to guaranteeing optimal performance of human genes in autologous expression systems. Despite individual reports describing mammalian expression enhancement using optimized genes (reviewed in [18]), no representative study has been carried out to scrutinize the general validity of improving autologous expression by gene optimization.

Here, we describe the first large-scale study addressing the influence of multiparameter optimization on autologous human protein expression. Our system was designed to represent the most important human protein classes. We provide evidence that our optimization approach is a reliable tool for improving expression, affecting processes at different molecular levels.

## Results

### Design of a comparative large-scale study on autologous expression of codon- and RNA-optimized human genes

To scrutinize the general validity of codon optimization for enhancing recombinant human protein expression in mammalian cell culture, we designed a large-scale study that included a broad selection of human genes. We chose 50 proteins from the NCBI-Entrez-database, representing the five most important protein classes of pharmaceutical and scientific interest: transcription factors (TF), ribosomal proteins (RB), protein kinases (PK), membrane proteins (MP), and immunomodulators (IM), summarized together with their database accession numbers in Table 1.

**Table 1:** Direct comparison of expression levels of 50 wildtype and sequence-optimized human genes.

Ref_seq	symbol	PC	length (bp)	CAI		% GC		codons altered (n)	codons altered (%)	ratio expression	expression statistics
				wt	opt	wt	opt			wt: opt	
NM_001514	**TFIIB**	TF	948	0.77	0.97	44%	62%	213	67%	**1∶1.18**	**+**
NM_004379	**CREB1**	TF	981	0.76	0.96	47%	63%	213	65%	**1∶2.80**	**+**
NM_014596	**ZNRD1**	TF	409	0.83	0.98	54%	64%	69	55%	**1∶3.41**	**+**
NM_016269	**Lef1**	TF	1197	0.82	0.97	51%	63%	232	58%	**1∶3.28**	**+**
NM_006331	**EMG1**	RB	763	0.79	0.97	51%	64%	149	61%	**1∶2.25**	**+**
NM_139071	**SMARCD1**	RB	1305	0.82	0.98	52%	62%	248	57%	**1∶1.85**	**+**
NM_002648	**PIM1**	PK	939	0.85	0.97	59%	62%	137	44%	**1∶1.12**	**+**
NM_006875	**PIM2**	PK	933	0.82	0.97	60%	65%	167	54%	**1∶1.28**	**+**
NM_001001852	**PIM3**	PK	978	0.87	0.97	69%	66%	130	40%	**1∶1.01**	** = **
NM_025195	**Trb1**	PK	1116	0.84	0.97	61%	64%	200	54%	**1∶1.23**	**+**
NM_004972	**JAK2**	PK	3396	0.77	0.98	39%	60%	784	69%	**1∶6.00**	**+**
NM_002110	**HCK**	PK	1578	0.88	0.98	57%	62%	224	43%	**1∶2.40**	**+**
NM_005356	**LCK**	PK	1527	0.87	0.98	58%	63%	222	44%	**1∶1.90**	**+**
NM_002019	**FLT1**	PK	4014	0.80	0.98	45%	60%	821	61%	**1∶3.94**	**+**
NM_002755	**MAP2K1**	PK	1179	0.84	0.97	53%	62%	207	53%	**1∶1.05**	** = **
NM_004073	**PLK3**	PK	1938	0.84	0.97	61%	64%	322	50%	**1∶1.15**	**+**
NM_002745	**Erk2**	PK	1080	0.81	0.98	47%	61%	204	57%	**1∶1.34**	**+**
NM_001315	**p38a**	PK	1080	0.82	0.98	47%	62%	213	59%	**1∶2.57**	**+**
NM_002750	**JNK1**	PK	1152	0.78	0.98	42%	60%	261	68%	**1∶2.78**	**+**
NM_002753	**JNK3**	PK	1266	0.81	0.98	45%	60%	238	56%	**1∶14.73**	**+**
NM_001292	**CLK3**	PK	487	0.85	0.97	60%	67%	92	61%	**1∶0.97**	** = **
NM_001892	**CK1a**	PK	1011	0.79	0.98	44%	60%	201	60%	**1∶1.95**	**+**
NM_003042	**GAT1**	MP	1797	0.87	0.98	56%	61%	254	42%	**1∶1.19**	**+**
NM_001045	Serotonin-TP*	MP	1890	0.83	0.97	53%	60%	322	51%	**1∶1.27**	**+**
NM_030956	**TLR10**	MP	2433	0.76	0.98	38%	58%	551	68%	**only opt**	**+**
NM_014437	**SLC39A1**	MP	972	0.83	0.96	62%	65%	160	49%	**1∶0.29**	**−**
NM_000220	**KCNJ1**	MP	1173	0.81	0.98	45%	60%	231	59%	**only opt**	**+**
NM_001651	**AQP5**	MP	795	0.87	0.96	63%	63%	112	42%	**1∶9.05**	**+**
NM_001753	**CAV1**	MP	534	0.84	0.99	50%	59%	88	49%	**1∶1.04**	** = **
NM_000593	**TAP1**	MP	2424	0.82	0.96	60%	65%	446	55%	**1∶1.97**	**+**
NM_000544	**TAP2**	MP	2109	0.84	0.96	59%	64%	345	49%	**1∶2.37**	**+**
NM_005561	**LAMP1**	MP	1251	0.84	0.97	56%	62%	206	49%	**1∶1.28**	**+**
NM_002294	**LAMP2**	MP	1230	0.78	0.98	43%	61%	280	68%	**only opt**	**+**
NM_014398	**LAMP3**	MP	1248	0.78	0.97	50%	64%	269	65%	**1∶2.80**	**+**
NM_000086	**CLN3**	MP	1314	0.84	0.97	60%	64%	211	48%	**1∶2.64**	**+**
NM_014319	**LEMD3**	MP	2733	0.76	0.96	52%	64%	583	64%	**1∶1.29**	**+**
NM_000914	**OPRM1**	MP	1200	0.83	0.98	49%	60%	212	53%	**1∶2.70**	**+**
NM_023921	**TAS2R10**	MP	921	0.75	0.99	36%	55%	204	66%	**only opt**	**+**
NM_024006	**VKORC1**	MP	520	0.83	0.98	59%	65%	73	45%	**1∶0.76**	**−**
NM_002507	**NGFR**	MP	1281	0.88	0.97	66%	66%	170	40%	**1∶1.15**	**+**
NM_000585	**IL-15**	IM	486	0.73	0.98	36%	58%	126	78%	**only opt**	**+**
NM_011337.2	**Mip1α**	IM	276	0.82	0.97	52%	59%	52	57%	**1∶1.48**	**+**
NM_000586	**IL-2**	IM	459	0.78	0.97	39%	58%	98	64%	**only opt**	**+**
NP_000610	**IFNγ**	IM	498	0.77	0.98	39%	56%	116	69%	**1∶1.81**	**+**
NM_006850	**IL-24**	IM	618	0.82	0.98	50%	62%	103	50%	**1∶1.20**	**+**
NM_000572	**IL-10**	IM	534	0.86	0.98	51%	59%	80	45%	**1∶1.35**	**+**
NM_002985	**RANTES**	IM	304	0.84	0.96	56%	62%	46	51%	**1∶1.04**	** = **
NM_024013	**IFNα**	IM	567	0.85	0.97	50%	61%	99	52%	**1∶1.16**	**+**
NM_001012271	**BIRC5**	other	495	0.84	0.96	54%	61%	84	51%	**1∶4.24**	**+**
NM_001786	**CDC2**	other	891	0.76	0.98	39%	59%	217	73%	**1∶2.85**	**+**

Columns left to right: Ref_seq.: Gene Bank accession number; symbol: encoded protein, e.g. ^*^Serotonin-TP  =  Serotonin transporter; PC: protein class (TF  =  transcription factor; RB  =  ribosomal protein; PK  =  kinase; MP  =  membrane protein; IM  =  immunomodulator; and two other proteins); length: size of open reading frame in base pairs; CAI (codon adaptation index) [19], [48]: measure for the “relative adaptability” of the codon usage of the gene of interest to codons used in highly expressed genes. The CAI represents the geometric mean of the relative adaptiveness values of the used codons. The relative adaptiveness value of a synonymous codon represents the ratio of the frequency of this codon in the codon usage of a given expression system and the frequency of the most frequent synonymous codon for the specific amino acid, leading to a value of 1.0 for the optimal codon and less frequently used codons are scaled down accordingly. %GC: percentage of GC-content in the respective transgene; codons altered (n): total number of codons altered in the open reading frame; codons altered (%): percentage of codons altered in the open reading frame; ratio expression wt: opt: mean expression from three independent transfections of wildtype genes were set to 1 and then compared to the mean expression of optimized genes; expression statistics: wt<opt: +; wt = opt (+/− 10%):  = ; wt>opt: −. Average variations ≥10% were considered an improvement in expression (**+**).

Using the sliding window approach [26] as described in the methods section we optimized the various candidate genes' coding regions taking the following sequence-based parameters into account (for review see [19]): *(i)* Codon choice, *(ii)* increase in GC-content, *(iii)* avoiding UpA- and introducing CpG-dinucleotides, *(iv)* removing destabilizing RNA elements, *(v)* removing cryptic splice-sites, *(vi)* avoiding intragenic poly(A)-sites, *(vii)* removing direct repeats, *(viii)* avoiding RNA secondary structures, and (*ix*) deleting internal ribosomal entry sites. All selected genes were synthesized *de novo* as wildtype and sequence-optimized versions, both encoding the same amino acid sequence. To assess protein expression, all coding regions were linked to a 3′-histidine_6_-tag to allow efficient detection using the α-Penta-His antibody. A FASTA file containing the sequences of all wildtype and sequence optimized constructs used in this study is provided as supplementary information (File S1).

### Gene optimization results in reliable expression and increased protein yields

**Figure 1:**
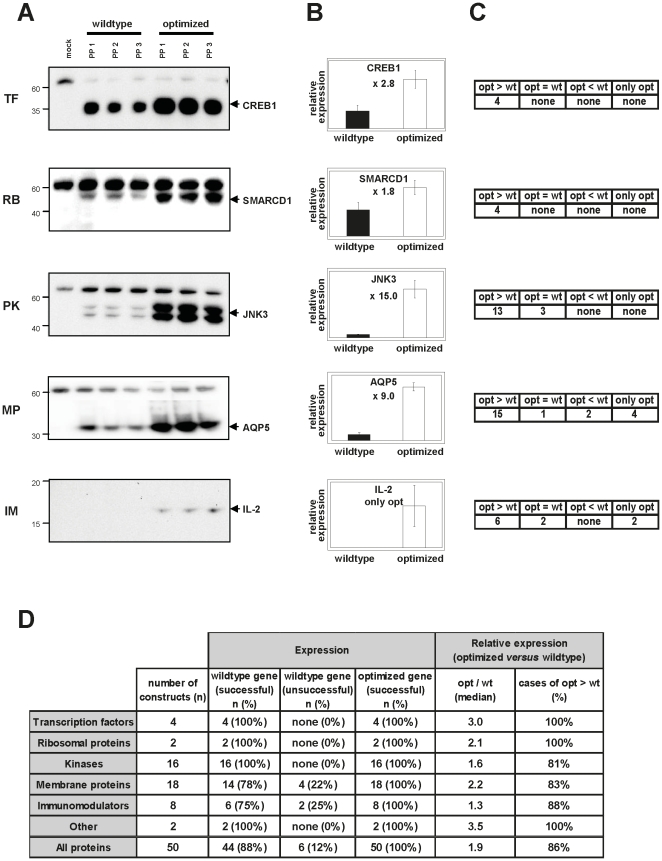
Comparative expression analysis of wildtype *versus* optimized genes representing different protein classes. (A) Each protein was expressed in triplicate (PP, plasmid preparation) in HEK293T cells. Either cell supernatants (immunomodulators, IM) or cell lysates (all other protein classes) were harvested and analyzed by Western blots using the α-Penta-His antibody. One example from each protein class is shown. A cross-reactive 60 kD band used to standardize protein amounts is visible, including in the empty vector negative controls (mock). Left: molecular weight markers, right: arrows indicating specific protein bands. (B) After quantifying Western blot signals, relative expression levels were derived from comparing mean expression (three independent transfections) of wildtype or optimized constructs, with wildtype set to 1 (see Table 1). The x-fold expression increase following gene optimization is indicated for each protein (only opt  =  no detectable wildtype expression). (C) Summary of relative expression levels of all proteins analyzed in each protein class. Average variations ≥10% were considered improved expression. (D) Statistical analysis of gene expression of (n) constructs in each protein class. Expression lists the number (n) and percent (%) of wildtype and optimized gene constructs expressed (successful) or not expressed (unsuccessful). Median opt/wt values of relative expression were calculated from total expression ratios derived as described above: opt/wt>1 indicates higher expression of optimized sequences. Where only the optimized construct was expressed, the opt/wt ratio was set to 2 for median calculation. Cases of opt>wt show the percentage of optimized constructs with elevated protein expression.

For statistical evaluation of gene expression, three different plasmid preparations of each construct were transfected independently into HEK293T cells. Equal sample amounts were analyzed by Western blotting, and signals were standardized against an endogenous 60 kD protein not affected by transgene expression, but reliably cross-reacting with the α-Penta-His antibody (Fig. 1A). Since three membrane proteins were not detected by the α-Penta-His antibody, we synthesized these genes with a 3×-Flag-tag, which enabled efficient detection of all six, wildtype and optimized, gene products. The respective protein amounts were standardized to endogenous GAPDH or β-actin levels. Commercial monoclonal antibodies were used for HCK- and LAMP1-specific protein detection (results not shown).

Sequence optimization frequently led to substantially elevated protein levels as seen in Western blots (Fig. 1A). Relative expression levels of wildtype and optimized gene constructs were calculated for each protein in all the protein classes (Fig. 1B, C; Table 1). Altogether, six out of 50 wildtype genes tested failed to express detectable levels of protein, whereas all 50 sequence-optimized constructs were successfully expressed (Fig. 1D; Table 1).

In summary, 96% of the optimized constructs performed equally, or better than their wildtype counterparts, while 86% clearly achieved increased protein expression levels. Notably, 53% of those 86% increased expression performance by at least 100% (Table 1), underlining the high quality of the *in silico* optimization and *de novo* synthesis process.

**Figure 2:**
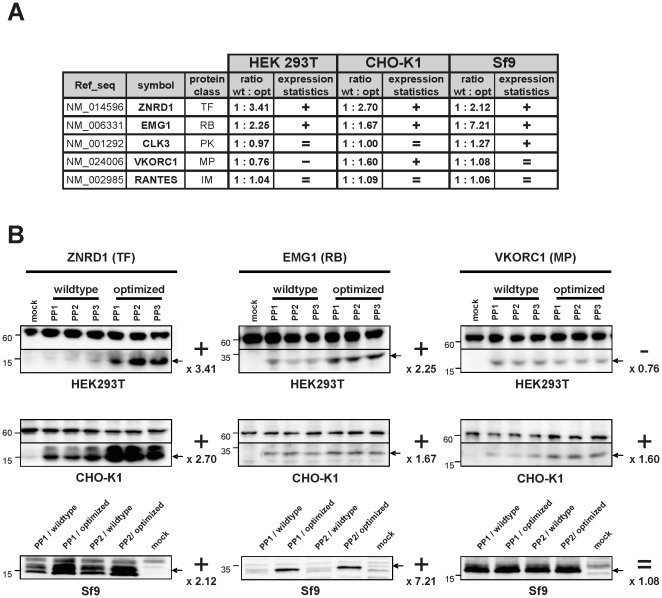
Comparative expression of human wildtype and human sequence-optimized gene constructs in HEK293T, CHO-K1 and insect-Sf9 cells. (A) Expression statistics for five representative proteins from each protein class. Mammalian HEK293T and CHO-K1 cells were transiently transfected in triplicate, whereas insect-Sf9 cells were transfected in duplicate. Relative protein expression of wildtype *versus* optimized genes (ratio wt:opt) was calculated from the mean expression values as described in Figure 1: (+) better expression of optimized gene; ( = ) comparable expression of both genes; (−) better expression of wildtype gene. (B) Western blot analyses of three representative proteins (panels left to right) transfected using three independent plasmid preparations (PP) into HEK293T and CHO-K1 cells, or two independent plasmid preparations into Sf9 cells (panels top to bottom). Signals from HEK293T and CHO-K1 cells were standardized against the ∼60 kD cross-reactive band serving as loading control (visible also in the mock negative control lane). Left: molecular weight markers, right: the x-fold increase (+), decrease (−) or equivalence ( = ) in expression of the optimized genes.

Next, we asked whether our optimization strategy is comparably efficient in other mammalian or eukaryotic cells. We tested five representative gene constructs from our collection in either CHO-K1, routinely used to generate stable cell lines, or insect-Sf9 cells widely used for recombinant protein production, in comparison to HEK293T cells (Fig. 2). In general, the impact of gene optimization was comparable in all three systems: All tested optimized constructs performed comparably, or even better than the wildtype genes in CHO or Sf9 cells, and only the optimized *vkorc1* gene was more poorly expressed in HEK293T cells. More importantly, these data demonstrate that the multiparameter algorithm used to optimize genes for mammalian expression is equally suitable for improving expression in insect cells.

### Gene optimization affects multiple levels of gene expression

To investigate the molecular mechanisms underlying optimization-based expression improvement, we chose the test gene *mip-1α*. This belongs to a family of cytokines subject to stringent and sensitive regulation, and might therefore be particularly susceptible to optimization-induced effects. To avoid potential saturation effects resulting from multi-copy expression in transient transfections, we generated cell lines expressing a stably integrated version of the wildtype or sequence-optimized *mip-1α* gene. The single-copy integration of the transgenes into a specific locus allows direct comparison of gene-specific effects in the same genomic context and should reveal minimal discrepancies in expression.

**Figure 3:**
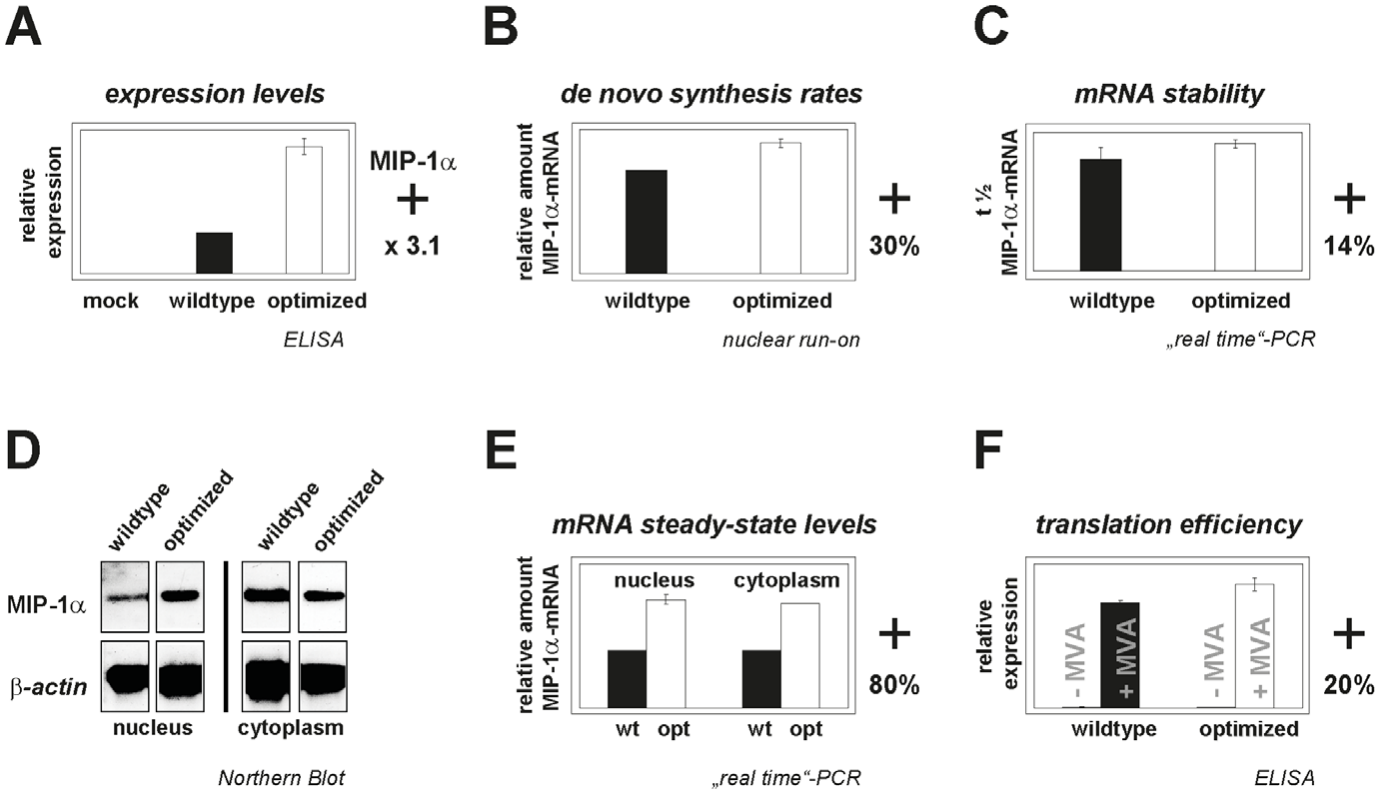
Influence of sequence optimization on expression-related mechanisms acting on stably integrated *mip1α* genes. (A) Relative protein expression levels of wildtype or optimized *mip1α* genes stably expressed in CHO-K1 cells were calculated from the mean values* measured by ELISA. (B) *De novo* transcription of RNA was measured by nuclear run-on assays. Cell nuclei were incubated with biotin-16-labeled dUTPs, separated via streptavidin-labeled magnetic beads, reverse-transcribed, and the resulting cDNAs were quantified by real-time PCR. *De novo* synthesized *mip-1α* transcripts* were normalized to *hph* cDNA levels, and the wildtype value* was set to 100%. (C) To determine mRNA stability both cell lines were incubated with 2.4 µM Actinomycin D for 0, 1.5, 3, 6, 12 and 24 hours. Total RNA was extracted at the respective time points and *mip-1α* mRNA levels quantified by real-time PCR were standardized against *hph-*specific mRNA amounts to obtain relative *mip-1α* mRNA half-lives of wildtype and optimized genes*. (D) Nuclear or cytoplasmic *mip-1α* mRNAs (2 µg) were subjected to Northern blot analysis using a DIG-labeled probe hybridizing to the BGH-polyA signal. Beta-actin served as an internal loading control. (E) Total RNA was separated from nuclear and cytoplasmic fractions, reverse-transcribed, and subjected to quantitative SYBR-Green real-time PCR using specific primers for both gene variants and the *hph* gene internal control. The resulting *mip-1α* cDNAs were verified by sequencing and amounts were standardized to *hph* cDNA levels to obtain mean mRNA steady-state values*. (F) To determine translation rates, HEK293T cells were infected with MVA-T7 prior to transient transfection with *mip-1α* variants under the control of a T7-promoter (+MVA). Transfected but uninfected cells served as negative controls (-MVA). Protein levels in cell supernatants were determined 24 hours post-transfection by ELISA. Expression levels obtained from wildtype transfections of infected cells were set to 100% and values from optimized genes were calculated accordingly. *Mean values derived from 2 independent experiments. **+** indicates relative improvements due to gene optimization.

MIP-1α production was determined by ELISA using culture supernatants, since secreted MIP-1α levels directly correspond to the expressed protein amounts. CHO cells expressing the optimized variant revealed a 300% increase in protein expression (Fig. 3A), a two-fold increase compared to MIP-1α expression in the transient HEK293T cell system (Table 1). It seems the single-copy status results in optimization-mediated effects becoming even more apparent in stably expressed genes.

Gene-specific effects on *de novo* RNA synthesis examined by nuclear run-on experiments revealed a 30% increase in RNA amounts transcribed from the optimized *mip-1α* gene (Fig. 3B). To test the influence of gene optimization on mRNA stability, we inhibited RNA synthesis with Actinomycin D for different time periods before determining *mip-1α* mRNA half-lives. Real-time PCR revealed that the optimized construct's mRNA half-life increased by 14% (Fig. 3C), suggesting gene optimization directly influences mRNA stability.

The combined positive effects of gene optimization on *de novo* synthesis rates and mRNA stability were expected to significantly increase the resulting mRNA steady-state levels. To confirm this, *mip-1α* transcripts isolated from nuclear and cytoplasmic cell fractions were analyzed by Northern blots (Fig. 3D). We detected a single distinct signal corresponding to the expected size of unspliced *mip-1α* mRNA, which argues against cryptic splicing events. We quantified mRNA amounts by reverse-transcription and quantitative real-time PCR of nuclear and cytoplasmic transcripts from both cell lines. The results confirmed previous observations, revealing an 80% increase in gene-optimized *mip-1α* transcript amounts in both cell fractions (Fig. 3E).

Finally, we tested the influence of gene optimization on translational efficiency using a cell-based translation assay. To exclude the nuclear compartment, HEK293T cells were infected with an MVA virus expressing a T7-RNA polymerase that mediates cytoplasmic transcription of transfected *mip-1α* genes under the control of the T7-promoter. MIP-1α levels were determined 24 hours post-transfection by ELISA (Fig. 3F). As expected, the optimized variant showed a 20% increase in translational efficiency, likely associated with the higher CAI value (Table 1).

Taken together, these experiments suggest that gene optimization affects gene expression at the transcriptional, posttranscriptional and translational level, thus significantly elevating MIP-1α protein levels.

### Kinases overexpressed from an optimized gene show unaltered activity

**Figure 4:**
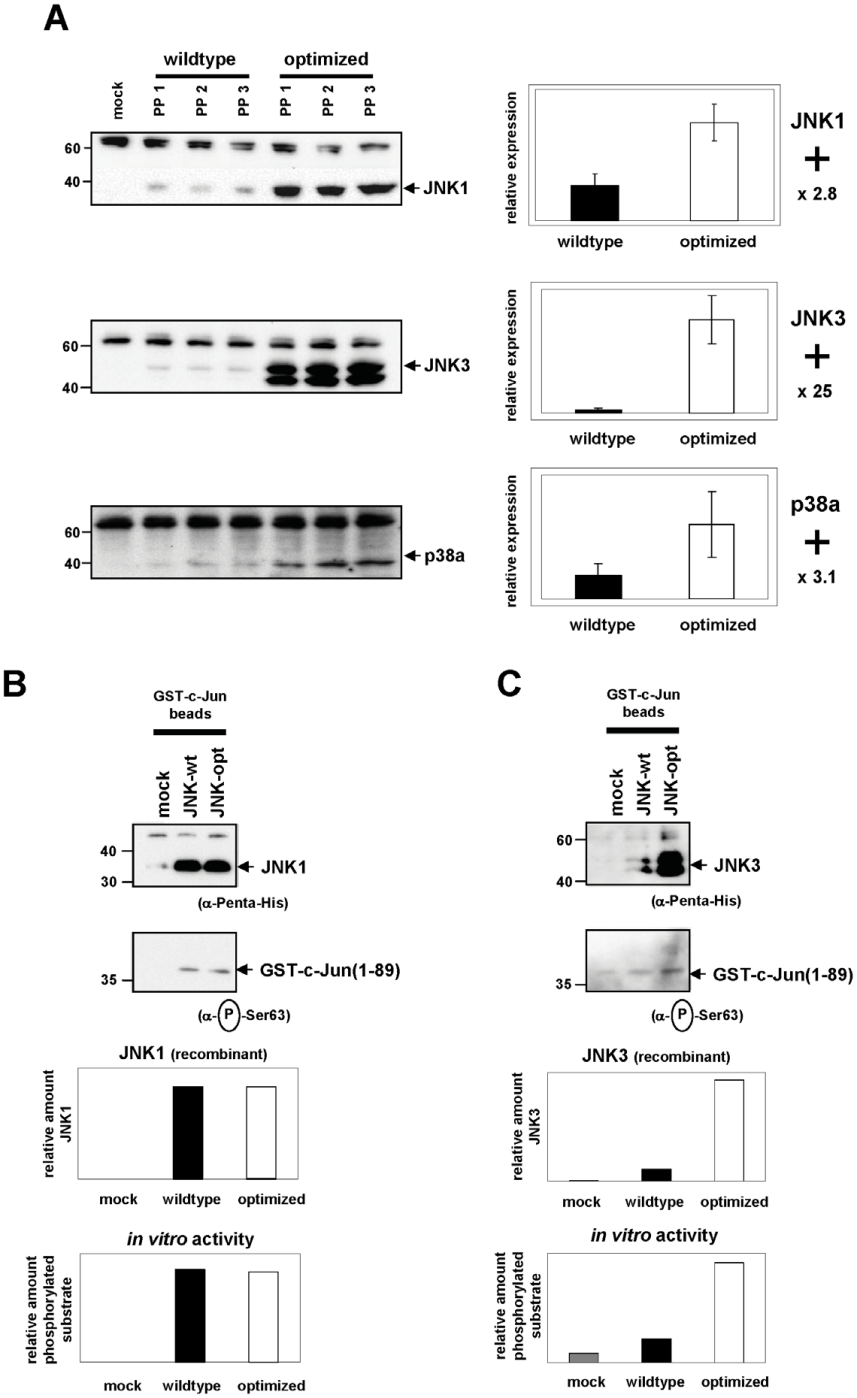
Solubility testing and *in vitro* analysis of JNK1- and JNK3 specific kinase activity. (A) HEK293T cells were transiently transfected with three different plasmid preparations (PP) of wildtype or optimized *jnk1, jnk3 and p38a-kinase* genes. Cells were lysed under mild conditions followed by subsequent centrifugation for 30 min at 16000 g and protein expression was analyzed by Western blots using the α-Penta-His antibody. Protein expression levels were standardized against the cross-reactive 60 kD protein band displayed on the blots. Relative expression was determined by relating the mean value obtained from optimized genes to the mean value of wildtype genes, with wildtype set at 1. (B) JNK1-kinase assay. Recombinant kinase proteins were purified from cell lysates and saturating amounts were pulled down with GST-c-Jun beads. Equal amounts of the protein complexes were subjected to Western blot analysis using the α-Penta-His antibody, JNK1 protein amounts in each sample were standardized against the cross-reactive 60 kD band. Kinase activity was quantified by *in vitro* phosphorylation of the bead-bound c-Jun substrate in the presence of ATP and subsequent detection of phosphorylated c-Jun proteins in Western blots using the antibody α-P-Ser63. (C) JNK3-kinase assay was carried out as described in (B).

Overexpression of transgenes in heterologous expression systems often results in insoluble and non-functional proteins due to misfolding or incorrect posttranslational modifications. In principle, autologous expression should overcome these problems, although sequence modifications introduced by gene optimization might influence protein folding, and therefore solubility and protein function. Given results of heterologous expression in *E. coli* showing that optimizing high level expression does not necessarily correlate with soluble protein production, we chose three kinases with significant expression level increase (JNK1 - 2.8-fold increase, JNK3 – 15-fold increase, p38a - 2.6-fold increase; Table 1) using heavy detergent lysis buffer. To test for solubility of overexpressed proteins, we reproduced protein expression but cells were lysed under more mild conditions followed by subsequent centrifugation for 30 minutes at 16000 g. Western Blot analysis confirmed our initial findings (Table 1) and resulted in even higher expression levels in case for JNK3 and p38a, demonstrating that overexpressed protein according to gene optimization was soluble (Fig. 4A). To compare the functionality of proteins produced from optimized or wildtype genes, we chose kinase JNK1 and JNK3 (showing the most significant expression level increase of all proteins in the study, Table 1, Fig.1) as representative candidates. JNK1 and JNK3 recombinant proteins purified under native conditions were incubated with GST-c-Jun-bound beads to test the capability of the respective kinases to phosphorylate their substrate. Western blotting of the kinase proteins pulled down by the GST-c-Jun beads confirmed equivalent (saturated) amounts of wildtype and optimized JNK1 (Fig. 4B, upper blot). *In vitro* phosphorylated c-Jun was then quantified by Western blotting using antibodies specific for phosphorylated substrate. No difference in *in vitro* activity was observed between the two kinase constructs, indicating that the increased expression induced by gene optimization had no impact on protein function (Fig. 4B, lower blot). In case for JNK3, Ni-affinity purification of only JNK3 “optimized” did provide sufficient protein to saturate GST-c-Jun substrate beads as demonstrated by Western blot analysis (data not shown), while “wildtype” JNK3 only bound minor amounts of protein (Fig. 4C, upper blot and panel) obtained from expression in a 6well format. Termination of the kinase reaction at a certain timepoint revealed recombinant kinase activity (wt AND opt) clearly over endogenous background activity (mock), while higher amounts of “optimized” protein resulted in higher amounts of phosphorylated substrate (Fig. 4C, lower blot and panel). This clearly demonstrates activity of overexpressed JNK3 kinase.

Recombinant kinase activity of p38a from optimized constructs was determined as well, resulting in *in vitro* phosphorylated substrate ATF-2, but could not be separated free of doubt from endogenous kinase activity (data not shown).

### Optimized synthetic genes represent valuable tools in RNAi

Short-interfering RNA (siRNA)-mediated gene silencing is a widespread strategy to analyze gene function. However, a key challenge is differentiating between a true cellular phenotype and so-called off-target effects, since a given siRNA may concomitantly trigger a multitude of unspecific secondary mechanisms. If siRNA-mediated downregulation of a specific gene provides a detectable cellular phenotype, a rescue experiment is required to see whether co-expressing the targeted gene with the siRNA restores the wildtype phenotype. Rescue experiments are often limited by the availability of siRNAs targeting the endogenous, but not the exogenous gene. Due to the presence of “silent mutations” in optimized genes, sequence-optimized constructs can be employed for virtually any RNAi rescue experiment.

**Figure 5:**
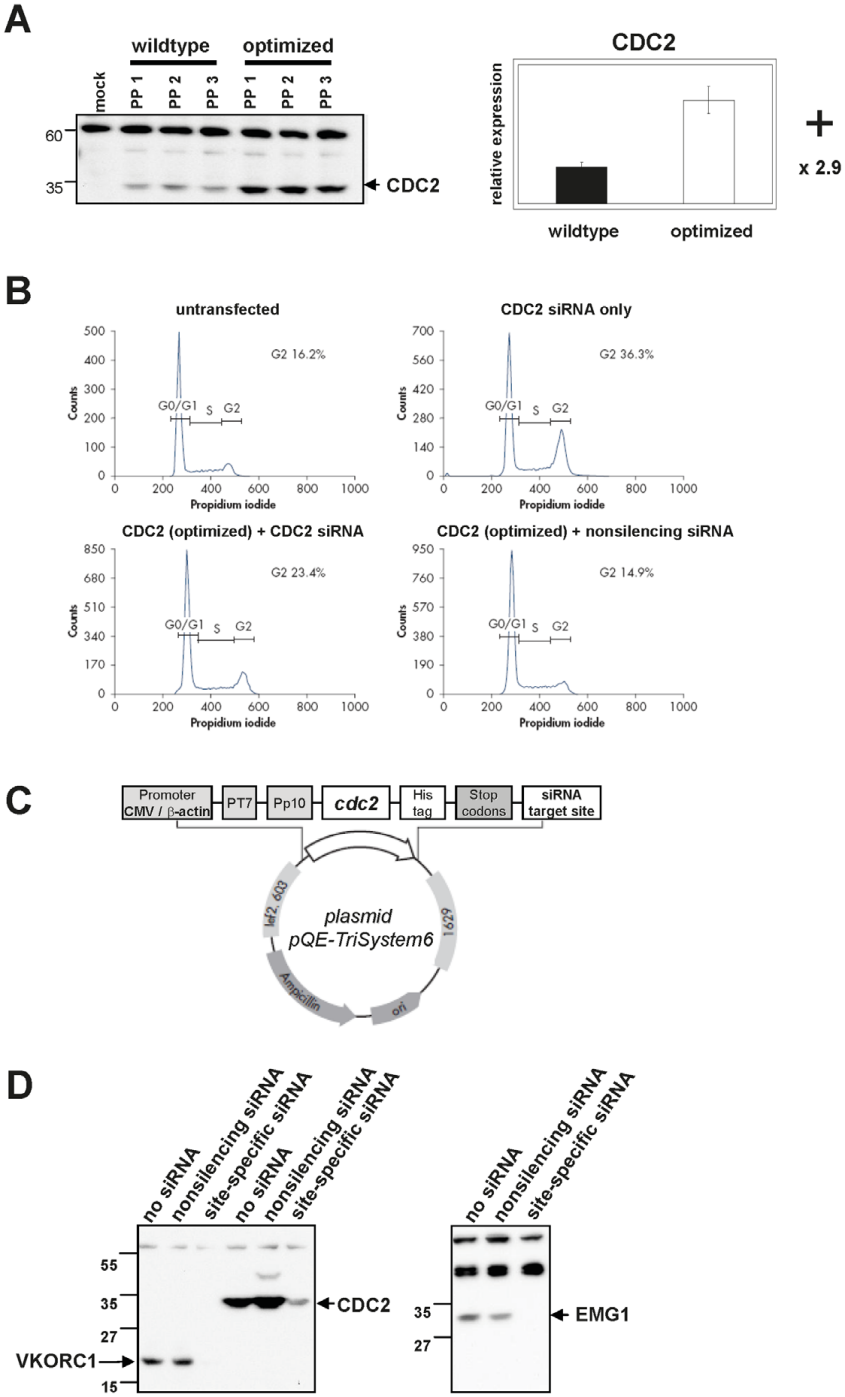
Rescue of siRNA-mediated knock-down of an endogenous gene with an optimized gene variant. (A) Cells were transiently transfected with three different plasmid preparations (PP) of wildtype and optimized *cdc2* genes and expression levels were analyzed by Western blotting using the α-Penta-His antibody. Relative expression was determined as described in Figure 1. (B) Untreated MCF-7 cells, or cells transfected with CDC2 siRNA only (knock-down), CDC2 siRNA plus the optimized *cdc2* gene (rescue), or a non-silencing siRNA plus the optimized *cdc2* construct were stained with propidium iodide after 72 hours and subjected to FACS analysis to determine cell-cycle distribution. The percentage of negative control cells compared to knockdown phenotype cells shifted from 16.2%/14.9% to 36.3%, i.e. around 20%. Negative control cells compared to rescued cells shifted from 16.2%/14.9% to 23.4%, i.e. around 8%, indicating that the optimized *cdc2* construct rescued around 60% of cells from knock-down. Endogenous CDC2 knockdown was confirmed by real-time RT-PCR with primers exclusively detecting endogenous *cdc2*, whereas expression of exogenous CDC2 from the sequence-optimized construct was confirmed by real-time RT-PCR with primers exclusively detecting exogenous *cdc2* (data not shown). (C) Schematic representation of the expression cassette in plasmid pQE-Tri-System6 containing the optimized *cdc2* gene sequence and the siRNA target site in the 3′ untranslated region. (D) The specificity of siRNA-mediated knockdown was tested by co-transfecting three sequence-optimized genes from different protein classes with site-specific or non-silencing siRNAs, followed by analyzing protein expression by Western blots.

To test this, we analyzed the cell cycle regulator CDC2 in MCF-7 cells, where the sequence-optimized gene construct expressed 2.9-fold higher protein levels than the wildtype (Fig. 5A). 16.2% of untransfected MCF-7 cells were in the G2 phase, as assessed by FACS analysis, but transfection of siRNA targeting endogenous *cdc2* mediated CDC2-knockdown to induce cell-cycle arrest, with 36.3% of the cells in the G2-phase (Fig. 5B). To verify that this cell-cycle arrest was CDC2-dependent, the sequence-optimized *cdc2* gene construct was co-transfected with CDC2 siRNA. Cells in the G2-phase were reduced to 23.4%, indicating that expression of the sequence-optimized CDC2 construct rescued around 60% of cells from the knockdown effect. Co-transfection of the sequence-optimized CDC2 construct with a non-silencing control did not affect cell-cycle distribution. Once again, the significantly increased expression of the sequence-optimized gene apparently did not influence protein function.

Occasionally, it might be desirable to silence or modulate the overexpression of a transgene. We tested the specific knockdown of three sequence-optimized constructs with an siRNA that does not target sequences in the human genome but specifically binds to a 3′ non-coding region present in the expression vector pQE-Tri-System6 (Fig. 5C). Satisfyingly, co-transfection of this unique siRNA mediated efficient downregulation of protein expression in all three cases tested (Fig. 5D). These results provide yet another example of how sequence-optimized constructs can be powerful tools in functional genomics.

## Discussion

Recent advances in gene optimization combined with *de novo* gene synthesis allow fast and efficient construction of synthetic genes individually tailored for specific applications. Whereas former approaches to optimizing genes or eliminating inhibitory motifs were mainly based on site-directed mutagenesis of a native template [15], [27], state-of-the-art techniques can rapidly synthesize full-length genes that have been sequence-optimized *in silico* based on the available amino acid sequence [19]. *De novo* synthesis has become affordable and guarantees controlled access to any of the 25,000 genes within the human genome, some of which are difficult to obtain by classic PCR-based cloning or have been incorrectly deposited in clone selection banks.

The simple sequence optimization strategy of backtranslating an amino acid sequence by using the most frequently used synonymous codon for each amino acid has been superseded by the development of advanced algorithms, which take into account multiple criteria to calculate a near optimal solution for the experimental requirements. Well-designed gene optimization is nevertheless a big challenge due to the fact that even a rather small amino acid sequence can result in a huge number of potential DNA sequences. The often employed Monte Carlo Methods take only a tiny fraction of the whole sequence space into account, and in most cases a less than optimal solution with respect to the theoretically ideal combination of codons representing the desired properties will be found in reasonable time. Many of the optimization parameters to be considered represent local sequence properties spanning a region of just a few dozen bases rather than global phenomena. This is obvious for codon usage, short sequence motifs, like restriction sites, splice site recognition patterns and other sequence elements but is also relevant regarding GC-content and the prevention of stable hairpin loops. Since it is unachievable to assess all possible codon combinations representing a given amino acid sequence, it becomes clear from the aforesaid, that it is acceptable for many sequence features to reduce the search space by performing an exhaustive search for the best solution only inside a small sequence window, which is moved along the whole reading frame. This sliding window approach [26], which was implemented in the GeneOptimizer® software and used for this study, has the additional advantage, that it performs unidirectional as sequences are processed naturally in the cell. Accordingly, the position dependent impact of certain sequence features, like the avoidance of bad codons near the 5′ end are taken into account properly [28], [29].

The effect of codon bias on expression has been analyzed for multiple individual genes. However, the focus remained on heterologous non-mammalian expression systems [18], [30]–[40]. Two multigene studies directly compared expression of 30 [30] and 100 [31] wildtype and sequence-optimized human genes in *E.coli*. Although optimized for *E.coli*, some human genes were still poorly expressed compared to their respective wildtype counterparts. Altogether, sequence optimization increased protein expression levels in *E.coli* for roughly 70% of expressible constructs [31] taking into account that a significant number of human proteins could not be expressed at all, possibly due to size or toxicity [30], [31].

Here, we provide evidence that improving autologous expression by multiparameter optimization can serve as a general strategy to overcome such difficulties. Although one might speculate that human genes need no optimization for autologous expression, most natural templates are “optimized” for maximum regulation rather than strong expression. Typical examples are transcription factors or cytokines, whose mRNAs display short half-lives in comparison to housekeeping genes [12], [34], or the highly regulated expression mechanisms of various human viruses, such as HIV, where codon optimization greatly benefits Rev-independent gene product expression [13], [14], [16], [41], [42].

All 50 sequence-optimized genes of our representative multigene study were successfully expressed under standardized conditions and at reproducible levels in different mammalian and insect cell lines. Consistent expression and yield are critical prerequisites for many downstream applications such as drug discovery, screening assays or biopharmaceutical production. This highlights a further advantage of autologous expression over the often unsuccessful expression of human genes in *E.coli* [31]. The majority of optimized genes induced a clear increase in detectable protein levels throughout all protein classes, while only two membrane proteins (VKORC1 and SLC39A1) were poorly expressed in HEK293T cells compared to their wildtype counterparts. We assume that this phenomenon is likely a cell-specific effect of overexpression rather than a direct result of optimization, since the respective genes showed comparable or even increased expression in CHO and insect-Sf9 cells. A more detailed sequence analysis comparing genes that were successfully optimized with those that were not, addressed CAI and GC content (Table 1), as well as CpG content, 5′CAI and ΔG values (data not shown) did not explain why 2 out 50 optimized genes showed decreased expression levels.

Increased expression triggered by codon-adaptation is mostly ascribed to translational effects [20], [43], [44], whereas more recent publications suggest that gene-optimization predominantly affects mRNA levels [24], [40]–[42], [45]–[47]. The results from cells stably expressing wildtype or optimized *mip-1α* genes demonstrate that our optimization approach affects expression on the transcriptional, posttranscriptional and translational level, while the secretory pathway was not affected by MIP-1α expression, according to only 1% of intracellular protein detected using the wildtype or optimized construct (unpublished data).

Gene-optimization significantly enhanced the CAI in all tested genes, a parameter often cited in the context of translational efficiency [17], [39], [48]. Accordingly, a high CAI correlated with clear improvement of MIP-1α translation as demonstrated in a cell-based assay. Interestingly, those wildtype genes showing no expression indeed mostly exhibit a relatively low CAI of ≤0.78 (Table 1), whereas all optimized genes mediating high-level expression have a CAI value close to 1, suggesting that the CAI might serve to predict the likelihood of successful expression in mammalian cells.

Apart from translation-specific effects, our gene-optimization clearly improved *mip-1α* mRNA steady-state levels and prolonged mRNA half-lives, correlating with a significant increase in GC-content. Although the GC-content appears to determine mRNA secondary structure and thus mRNA stability, it cannot account for the overall improvement in expression achieved by the optimized genes, since some of them display a GC-content similar to their wildtype counterparts. A strong increase in mRNA levels has been described for individual genes using the same gene-optimization approach [10], [16], [17], [19], [31]. However, it remains to be determined in individual cases to what extent enhanced mRNA structure/stability or increased *de novo* transcription, as specifically demonstrated for the optimized *mip-1α* gene, contribute to the available RNA amounts. The latter observation is particularly interesting due to a recent publication assigning a role to intragenic CpG-dinucleotides in boosting transcriptional activity [10]. This hypothesis would underline the importance of codon composition and the contribution of specific-sequence motifs to overall protein production. The sequence determinants driving optimal performance in mammalian cells are presumably far more complex than those affecting expression in bacterial hosts, which – apart from codon bias – seems to strongly depend on the stability of 5′mRNA structures [38], [40]. A recent report even suggests that codon order, and correlation with isoaccepting-tRNAs, rather than codon composition, contribute to rapid translation in eukaryotes [49].

These insights will certainly help to adapt and improve future optimization strategies for maximum expression success. Notwithstanding, this large-scale study proves that our multiparameter optimization was successful with 50 human genes representing the most important protein classes. Gene optimization clearly improved protein expression in the majority of cases and selected overexpressed gene products proved to be functional.

In principle, one would assume that autologous expression should overcome problems of overexpression such as insolubility or misfolding of proteins resulting in non-functional protein as often observed for heterologous expression systems such as *E. coli*. Nevertheless, sequence modifications introduced by gene optimization might influence protein folding, and therefore solubility and/or function. However, potentially insoluble or non-functional protein due to overexpression is not a problem of gene optimization per se, and functionality and solubility has to be analysed for each case of overexpressed protein and any “expression optimization strategy”, such as e.g. the use of strong promoters, integration copy number, fermentation conditions, etc. Our results are very encouraging, since high expressers with an expression level increase of 2.6-fold to 15-fold showed no detrimental effect on solubility (JNK1, JNK3, p38a) or function (JNK1, JNK3 and CDC2). This positive effect of gene optimization on protein expression resulting in functional protein was also demonstrated in a recent publication by some of the authors [50], where a single electro-gene transfer of an RNA- and codon optimized EPO gene into skeletal muscle resulted in a 3- to 4-fold increase of EPO production over mice treated with non-optimized EPO genes, sustaining for >1 year and triggering a significant increase in hematocrit and hemoglobin without causing adverse effects [50]. Furthermore in addition to the mechanistic insights of overexpression in the stable system described for MIP1-α, the study provides supporting mechanistic insights of overexpression in a transient system [50].

Finally, particularly interesting, the successful application of optimized genes in RNAi experiments emphasizes the potential and value of gene optimization in functional genomics research. We belief that *de novo* synthesis of RNA- and codon-optimized genes will become a standard process for recombinant human protein production, and will serve to improve and standardize any application relying on reproducible, efficient and high quality expression.

## Materials and Methods

### Construct design and optimization

Human gene sequences were obtained from the NCBI GeneEntrez Database. The coding regions were optimized using the GeneOptimizer® expert software, employing a deterministic sliding window algorithm [26] to cope with the vast sequence space in multiparameter DNA sequence optimization. A variation window covering several amino acid positions slides along the coding sequence. Candidate sequences are built comprising a section of the already optimized sequence upstream to the variation window and each of all possible combinations of synonymous codons within the window. The candidate sequences are assessed with a quality function [26] taking codon usage, GC-content, mRNA structure and species-specific sequence motifs into account. The first codon of the best candidates' variation window is fixed and the window is shifted by one codon position towards the 3′end.

Wildtype and sequence-optimized genes were synthesized using synthetic oligonucleotides, assembled by primer extension-based PCR, cloned, and verified by sequencing (for review see [19] page 425–438). All constructs contain a C-terminal His_6_-tag followed by two STOP-codons to ensure efficient termination. *Slc39A*, *cln3*, and *serotonin-tp* genes were synthesized as wildtype and optimized versions containing a Flag_3_-tag separated by a serine-glycine-linker.

### Cell culture and protein expression

For expression in mammalian or insect cells, wildtype and sequence-optimized transgenes were cloned into plasmids pQE-TriSystem6 (Qiagen) or pIEx-4 (Novagen). After preparing three independent plasmid preparations from separate clones, 1.2 µg of vector DNA was transiently transfected into HEK293T (HEK 293T/17, ATCC, *CRL-11268* **)** and CHO cells (CHO-K1, ATTC, *CCL-61*) seeded at 80-90% density, using Attractene (Qiagen) or Fugene (Roche) according to the manufacturer's instructions in OPTI-PRO serum-free medium (Invitrogen). Insect-Sf9 cells (Novagen, *Cat.-No.∶71104*) were transfected using GeneJuice (Novagen). Cell lines stably expressing MIP-1α constructs were generated using the Flp-In System (Invitrogen) according to the manufacturer's instructions. Constructs were cloned into vector pcDNA5/FRT (Invitrogen) and transfected into CHO Flp-In-cells (Invitrogen). Positive clones were selected with increasing amounts of hygromycin B at a maximum concentration of 500 µg/ml.

### Protein expression analysis

Transfected HEK293T and CHO cells were harvested after 2–3 days in TDLB buffer (50 mM Tris/HCl pH 8.0; 150 mM NaCl; 0.5% sodium deoxycholat; 0.1% SDS; 0.1% TritonX-100) and sonicated (Bandelin Sonoplus, cycle 5). Kinases tested for soluble protein were harvested in 20 mM Tris (pH 7.5), 150 mM NaCl, 1 mM EDTA, 1 mM EGTA, 1% Triton, 2.5 mM sodium pyrophosphate, 1 mM β-glycerophosphate,1 mM Na3VO4, 1 µg/ml Leupeptin, sonicated and centrifuged for 30 minutes at 16000 g.

Immunomodulators were precipitated with TCA from harvested cell supernatants. Protein expression was quantified as described earlier [26]. Protein concentration was measured using DC Protein Assay (Bio-Rad) and equal amounts were loaded on 4–20%-SDS–PAGE-gels (Invitrogen) for Western Blot analysis. Western Blot signals were detected using α-Penta-His antibody (Qiagen) with BM Chemiluminescence Western-Blotting-Substrate (POD) (Roche) or SuperSignal West-Femto-Maximum-Sensitivity-Substrate (ThermoScientific) and quantified using GelProAnalyzer-Software6 (INTAS). Wildtype and optimized constructs were analyzed in triplicates on the same gel, by measuring the integrated optical density (IOD) of each protein signal in the linear range of a 16 bit CCD camera system. In contrast to the low dynamic range and fast saturation on X-ray film, no saturation effects were detected in any measures. Expression levels were standardized against an endogenous 60 kD cross-reactive band by measuring the integrated optical density (IOD) of each band. Quantified results were standardised, averaged and the ratio wildtype (set at 100%) versus optimized construct was determined. Lysate from mock-treated cells, transformed with the empty expression construct, served as negative controls for analysis. Flag-tagged proteins or proteins detected with specific antibodies were standardized against endogenous GAPDH or β-actin as described above. Proteins expressed in Sf9 cells were quantified using fluorescence-based methods as described elsewhere [31]. Expression levels of stably integrated *mip-1α* genes were measured using a commercial ELISA kit (R&D Systems).

### RNA analysis

Northern blot analysis was performed as described earlier [10]. Nuclei and cytoplasm were separated by centrifugation, and RNA was isolated using the RNeasy-Kit (Qiagen). Specific mRNAs were detected via chemiluminescence using Digoxigenin (DIG)-labeled probes and α-DIG-antibodies (Roche). MIP-1α-antisense RNA probes hybridizing to the BGH-polyA signal present in all transcripts were generated using the “Riboprobe *in vitro* Transcription Kit” (Promega). For *in vitro* transcription a T7 promoter-extended PCR product was generated, enabling initiation of T7-polymerase. DIG-11-UTP was incorporated for detecting the probe; *mip-1α* probe: 5′-CTCGAGCATGCATCTAGAGGGCCCTATTCTATAGT GTCACCTAAATGCTAGAGCTCGCTGATCAGCCTCGACTGTGCCTTCTAGTTGCCA GCCATCTGTTGTTTGCCCCTCCCCCGTGCCTTCCTTGACCCTGGAAGGTGCCACTC CCACTGTCCTTTCC-3′; β-actin-probe: 5′-AGAGGCATACAGGGACAGCACAGCCTG AATGGCTACGTACATGGCTGGGGTGTTGAAGGTCTCAAACATGATCTATAAAGA AAAATGAGGCATTGTCAAACTCCAAAAGCCACAAGTAGTCAAGGCAGGTAGGAC TGTCAGGACAGATATGGGACATGCAGAGTGCAAGAACACAGCTAAGGTAAGTGT GCTGGGAGAAATCTCAGGACAGGGGCTCCATTTTAAACCTACTGTGCATCTACTGAATACACACTCCAAGGCCACTTATCACCAGCCTCAT-3′

### Real-time PCR

Total RNA was extracted as described above. cDNA was synthesized using oligo(dT)_15_ primers, M-MLV RNaseH-PointMutant reverse transcriptase (Promega) and 500 ng DNA-free RNA as templates according to manufacturer's guidelines. Reverse-transcribed RNA was quantified using DyNAmo Capillary SYBR Green qPCR kits (Finnzymes) as described earlier [51]. Forward and reverse oligonucleotides for amplifying the entire open reading frames were 5′-ATGAAGGTCTCCACCACTGC-3′ and 5′-TCATGAAGACTAGGCATTCAG TTC-3′ for wildtype *mip-1α*, 5′-ATGAAGGTGAGCACCACAGCT-3′ and 5′-TCATGAA GACTAGGCGTTCAGC-3′ for optimized *mip-1α*, and 5′-CTGGAGCGAGGCGATGTTC-3′ and 5′-CTGCGGGCGATTTGTGTAC-3′ for the *hph* gene.

PCR efficiency of the respective oligonucleotides was analyzed using serial plasmid dilutions and determined to be 1,847 for *mip1α* wildtype and 1,828 for the optimized *mip1α* gene. Real-time PCR derived data were quantified relatively according to Pfaffl *et al.* [51] taking the divergent efficiencies into account. The specificity of obtained PCR products was verified via melting curve analysis and sequencing.

### Nuclear run-on and mRNA half-life

Nuclear run-on analysis was performed as formerly described [52], using biotin labeling, magnetic bead capture and analysis by fluorescence-based RT-PCR. *De novo* synthesized RNA was quantified using real-time PCR as described above. mRNA half-life was analyzed as described in Leclerc et al. [53].

### MVA-T7-mediated expression

For cytoplasmic *mip-1α* expression under the control of the T7-promoter, HEK293T cells were infected at an MOI of 10 with modified Vaccinia-Ankara virus providing a T7-RNA polymerase (MVA-T7) followed by transient transfections with vector pPCR-Script (pT7, Stratagene) containing the *mip-1α* genes under the control of a T7-promoter. MIP-1α levels were determined 24 hours post-transfection by ELISA.

### Kinase assay

Cell lysates of cells transfected with wildtype or optimized *jnk1-* and *jnk3*-constructs were prepared in triplicates according to a commercial assay protocol (SAPK/JNK-Assay-Kit (Nonradioactive), Cell Signaling Technology®). Samples were pooled, adjusted to 20 mM imidazole and purified with 20 µl of Ni-agarose beads to remove endogenous kinase activity (HIS-Select™ Nickel Affinity Gel, Sigma). Ni-bound proteins were washed (PBS, 500 mM NaCl, 20 mM imidazole) and eluted for 30 min at room temperature (PBS, 500 mM NaCl, 200 mM imidazole). Saturating amounts of eluted protein were pulled down with GST-c-Jun-coated beads and kinase activity was determined in the presence of ATP according to the above protocol.

### Gene silencing and rescue

To knock-down endogenous CDC2, MCF-7 cells (DSMZ, *DSMZ no.∶ ACC115*) were transfected with 50 nM of a *cdc2*-specific siRNA using HiPerFect (Qiagen). CDC2 knock-down was rescued by co-transfecting 0.4 µg of the pQE-TriSystem6 vector carrying a sequence-optimized *cdc2* gene. To determine cell-cycle distribution, cells were harvested 72 hours post-transfection, stained with propidium iodide and subjected to FACS analysis.

For siRNA-mediated gene silencing, 1 µg of vector pQE-TriSystem6 DNA encoding sequence-optimized genes (Fig. 5C) was co-transfected with 8.4 nM of an siRNA targeting the 3′ untranslated region 5′-AAGCGTTGAAATAGCGTACAA-3′ of the expression construct. Cells were harvested 48 hours post-transfection and analyzed by Western blotting using the α-Penta-His antibody.

## Supporting Information

File S1 Construct sequences. File S1 contains the sequences of all wildtype and sequence optimized constructs used in this study.
(FAS)
