# Correction: Multiparameter RNA and Codon Optimization: A Standardized Tool to Assess and Enhance Autologous Mammalian Gene Expression

**DOI:** 10.1371/annotation/039deb02-bbe7-406c-a876-341cc4f3fefa

**Published:** 2011-03-21

**Authors:** Stephan Fath, Asli Petra Bauer, Michael Liss, Anne Spriestersbach, Barbara Maertens, Peter Hahn, Christine Ludwig, Frank Schäfer, Marcus Graf, Ralf Wagner

The authors would like to update the first affiliation. "Geneart AG, BioPark, Regensburg, Germany" should now be: "Geneart/Life Technologies, BioPark, Regensburg, Germany."

